# Pelvic floor physical therapy in patients with chronic anal fissure: a randomized controlled trial

**DOI:** 10.1007/s10151-022-02618-9

**Published:** 2022-05-05

**Authors:** Daniëlle A. van Reijn-Baggen, Henk W. Elzevier, H. Putter, Rob C. M. Pelger, Ingrid J. M. Han-Geurts

**Affiliations:** 1 Department of Surgery, Proctos Clinic, Bilthoven, The Netherlands; 2 Department of Urology and Medical Decision Making, Leiden University Medical Centre, Leiden, The Netherlands; 3 Department of Urology, Leiden University Medical Centre, Leiden, The Netherlands; 4 Department of Biomedical Data Science, Leiden University Medical Centre, Leiden, The Netherlands

**Keywords:** Chronic anal fissure, Anal pain, Treatment, Pelvic floor physical therapy, Biofeedback

## Abstract

**Background:**

A chronic anal fissure is a common, painful condition with great impact on daily life. The exact pathogenesis has not been fully elucidated and treatment varies. A large percentage of patients experience pelvic floor dysfunction (dyssynergia and increased pelvic floor muscle tone). The aim of our study was to investigate the effect of pelvic floor physical therapy in patients with chronic anal fissure.

**Methods:**

Between December 2018 and July 2021, at the Proctos Clinic in the Netherlands, patients with chronic anal fissure and pelvic floor dysfunction were randomly assigned to an intervention group, receiving 8 weeks of pelvic floor physical therapy including electromyographic biofeedback or assigned to a control group receiving postponed pelvic floor physical therapy. The primary outcome was muscle tone at rest during electromyographic registration of the pelvic floor before and after pelvic floor physical therapy. Secondary outcomes contained healing of the fissure, pain ratings, improvement of pelvic floor function, and complaint reduction measured with a proctology-specific patient-reported outcome measurement. Endpoints were measured at 8- and 20-week follow-up.

**Results:**

One hundred forty patients were included in the study, 68 men (48.6%) and 72 women (51.4%) with a mean age of 44.5 ± 11.1 (range 19–79) years. Mean resting electromyographic values of the pelvic floor in the intervention group significantly improved from pre- to post-treatment (*p* < 0.001) and relative to controls (mean estimated difference between groups − 1.88 µV; 95% CI, − 2.49 to − 1.27 (*p* < 0.001) at first follow-up and remained significant from baseline at 20-week follow-up *(p* < 0.001). The intervention group performed better compared to the control group on all secondary outcomes, i.e., healing of the fissure (55.7% of the patients vs 21.4% in control, pain ratings (*p* < 0.001), diminished dyssynergia (*p* < 0.001), complaint reduction (*p* < 0.001), and decrease of pelvic floor muscle tone (*p* < 0.05) at first follow-up.

**Conclusions:**

The findings of this study provide strong evidence that pelvic floor physical therapy is effective in patients with chronic anal fissure and pelvic floor dysfunction and supports its recommendation as adjuvant treatment besides regular conservative treatment.

## Introduction

### Background and objectives

Chronic anal fissure (CAF) is one of the most common proctological problems. It causes significant morbidity and has a large impact on quality of life [1, 2]. An anal fissure refers to a longitudinal ulcer in the squamous epithelium, generally located in the posterior midline [3]. The classical symptom is pain during defecation, which may persist for hours [3, 4].

The exact pathogenesis of CAF is debatable. Passing of hard stools or sudden evacuation of liquid stool can lead to mucosal damage, resulting in an overreaction of the external anal sphincter (EAS] continence reflex and an increase of basal resting pressure. This could lead to spasm, thus leading to reduced blood flow and ischemia, which prevents CAF from healing [5–8].

Defecation is a complex function. Normal defecation requires anorectal synchronization, an intact rectal sensation and perception, a contraction of the abdominal muscles, and relaxation of the EAS and puborectalis muscle. To evacuate stool, it is essential that the puborectalis muscle relaxes for straightening the anorectal angle [9]. When the pelvic floor muscles do not relax or even contract (dyssynergia) during attempted defecation, this could result in an increase in the anorectal angle and hence prohibits the normal passage of stool [10]. Dyssynergia and increased pelvic floor muscle tone are likely to be factors contributing to delayed healing and pain in patients with CAF [11, 12].

Initial treatment of CAF is based on conservative management with fiber and/or laxatives to alleviate constipation. Treatment with ointment is directed toward relieving internal sphincter spasm, thus improving circulation and pain relief [13]. If unresponsive to conservative management including ointment, botulinum toxin injections may be considered; however, this is associated with recurrence rates of 18–50% [3, 14, 15]. Another option and currently the gold standard of surgical intervention is lateral internal sphincterotomy [16]. Nevertheless, its potential risk of causing incontinence, 3.4–14%, should be kept in mind when considering this treatment [14, 16–18].

In patients with CAF, who have also been diagnosed with pelvic floor dysfunction, pelvic floor physical therapy (PFPT) may add to adequate treatment. The aim of PFPT is to increase awareness and proprioception, to restore abdominopelvic coordination, to improve muscle relaxation and elasticity of the pelvic floor, and reduce pain [19, 20]. PFPT including biofeedback therapy has already been proven effective in the treatment of increased pelvic floor muscle tone and dyssynergia [19, 21–24], but has not been investigated in patients with CAF.

We hypothesized that treatment with PFPT including biofeedback in addition to regular conservative management will result in an improvement of pelvic floor muscle tone and function, pain, healing of the fissure, and increased satisfaction in patients with CAF and concomitant pelvic floor dysfunction.

## Materials and methods

### Study design

The PAF study is a single-center, parallel, randomized controlled trial. This superiority trial was designed to detect a difference of PFPT including surface electromyographic biofeedback (EMG) versus no PFPT at first follow-up. The design involved allocation of all appropriate consecutive patients with CAF and pelvic floor dysfunction. Eligible patients were randomly assigned, after providing written informed consent, to an intervention group receiving 8 weeks of PFPT including EMG-biofeedback, or assigned to a control group receiving postponed PFPT.

### Baseline and follow-up

Baseline and follow-up appointments at 8 and 20 weeks from baseline with the surgeon and principal investigator, an experienced pelvic floor physical therapist, consisted of a clinical examination provided through inspection to investigate the healing of the fissure. If necessary, proctoscopy was performed to exclude other pathology. Resting anal sphincter pressure, and pelvic floor muscle tone and function were measured by a careful digital rectal examination and scored as decreased, normal, and increased [25, 26]. Pelvic floor dysfunction was defined by the presence of dyssynergia and/or increased pelvic floor muscle tone. Besides that, pelvic floor muscle tone was measured with EMG (μV) [25] with an intra-anal probe (Maple,®Novuqare Pelvic Health B.V. CE 0344, Rosmalen, The Netherlands). This probe has a matrix of 24 electrodes and is capable of registering EMG-activity nearest to the individual muscles of the pelvic floor during diagnosis and treatment. The Maple® system is validated for its purpose [27]. In addition, muscle tone of the EAS was measured with EMG (circle 1, Maple®).

Dyssynergia was detected by digital rectal examination [28] and balloon expulsion test [29]. The balloon expulsion test provides an assessment of the patient’s ability to evacuate artificial stool during simulated defecation. A non-sterile disposable balloon (BARD, Covington, USA) was filled with 50 ml water or until the patient felt an urge to defecate. Evacuation of the balloon after more than 2 min was seen as impossible to expulse and was considered dyssynergic defecation [29]. The balloon expulsion test was performed at baseline and 20-week follow-up by the nurse in our clinic.

Patients were requested to fill in 2 validated self- administered questionnaires at baseline, and at 8- and 20-week follow-up. To quantify the average intensity of pain during defecation, a visual analog scale (VAS) from 0 (no pain) to 10 (most intense pain) was used [30]. The Proctoprom, a patient-related outcome measurement was used to assess the impact of proctologic complaints on different aspects of a patient’s life and to evaluate the effect of treatment [31].

### Participants

Men and women aged 18 years or older presenting CAF and pelvic floor dysfunction were recruited at the Proctos Clinic in the Netherlands from December 2018 until July 2021. CAF was defined as a longitudinal ulcer with symptoms presenting longer than 6 weeks or recurrent fissures.

All patients had failed conservative treatment with fiber and/or laxatives and ointment (diltiazem or isosorbide dinitrate) used for at least 6 weeks and with accurate instructions about how to apply. All patients had sufficient understanding of the Dutch language (reading and writing) and were able to complete online questionnaires. We considered patients who were not able to undergo a digital rectal examination, not eligible for this study. Patients with an abscess or fistula, Crohn’s disease or ulcerative colitis, anorectal malignancy, prior rectal radiation, and pregnancy were excluded from the study.

### Interventions

At baseline, patients in both groups received information about the pelvic floor and related symptoms, explanations about relevant anatomy and defecation (patho)physiology, behavioral modifications, and lifestyle advice. All patients continued their conservative measures including the use of ointment (diltiazem or isosorbide dinitrate).

PFPT consisted of 5 face-to-face appointments of a mean of 45 min in a period of 8 consecutive weeks, using a treatment protocol [32]. Patients were referred to an extra-mural private practice, preferably nearby patients’ home address.

The treatment protocol was comprised of intrarectal myofascial techniques, such as stretching the puborectalis muscle and myofascial release on identified trigger points in the pelvic floor to increase flexibility, release muscle tension, and improve circulation. Manual techniques were tailored to the patient and based on results and findings of the diagnostic evaluation of the pelvic floor at every visit. To gain awareness, patients were taught how to contract and relax the pelvic floor muscles and were learned how to incorporate these into daily life. Breathing and pelvic floor muscle exercises were combined with EMG-biofeedback with an intra-anal probe (Maple®) [27]. The sessions were performed to increase awareness and monitor pelvic floor (dys)function [19, 20]. Patients with pelvic floor dyssynergia learned how to relax the pelvic floor during straining. If patients were unable to contract or relax the pelvic floor muscles, neuromuscular electrical stimulation was applied intra-anally during the biofeedback session. The home exercise program incorporated stretching the puborectalis muscle during the application of prescribed ointment, and pelvic floor muscle and breathing exercises to improve relaxation. Furthermore, patients used thermotherapy with a heat blanket or sitz baths for relaxation [33]. Additionally, information was provided with folders and videos to guide the home exercises.

Patients who were assigned to postponed PFPT did not receive additional treatment besides their conservative measures until first follow-up at 8 weeks after inclusion.

All medical data were collected at the clinic before entry into the trial database, and data collection was facilitated by case record forms in Castor EDC [34]. We recorded all adverse events and serious adverse events.

### Outcome measures

The primary outcome was muscle tone at rest during EMG-registration of the pelvic floor before and after PFPT.

Secondary outcomes contained clinical healing of the fissure (complete re-epithelisation), average pain intensity during defecation on a VAS-scale, improvement of pelvic floor muscle function, and complaint reduction measured with the Proctoprom before and after PFPT.

All outcomes were measured at baseline, at 8- and 20-week follow-up.

### Sample size

The sample size of the study was based on the primary outcome of the study, the tone at rest during EMG-registration of the pelvic floor. In preliminary studies, we found a mean of 1.75 (μV) at rest, with a standard deviation of 1.75. Based on a slightly conservative standard deviation of 1.8, and a difference to be detected of 1.0 between the treatment group and the control group, we concluded that at least 70 patients in each treatment arm were required to detect a difference of 1.0 between the treatment group and the control group with postponed treatment. This sample size provided ample power (> 90%) to detect a moderate-effect size with a nominal alpha level of 5%.

### Randomization

The surgeon and the principal investigator approached the patient and informed the patient about the study. Patients who met the eligibility criteria were randomly assigned to the PFPT treatment group or to the control group receiving postponed PFPT (1:1 allocation, random block sizes of 4, 6, and 8). The randomization was computer generated using Castor EDC [34]. A unique record number was generated, and the allocation was disclosed. The principal investigator was not able to access the randomization sequence and had a decoding list with randomization numbers and patient identification numbers in the investigator site file. Only the coordinating surgeon and principal investigator had access to the key to the code. The principal investigator informed the patient about group allocation and follow-up appointments.

### Blinding

The principal investigator who was also involved in the data analysis was not blinded for allocation. Because of the nature of the intervention, the principal investigator, collaborating pelvic floor physical therapists and patients could not be blinded. However, the surgeon performing the 8- and 20-week follow-up to investigate the healing of the fissure, resting anal sphincter pressure, and pelvic floor dyssynergia was blinded to group allocation.

### Statistical analysis

Data were analyzed using Statistical Packages for Social Sciences (SPSS, Chicago, II, USA, version 26.0). Descriptive methods were used to assess quality of data, homogeneity of treatment groups, and endpoints. Normality of the data were analyzed with histograms. Data are presented using mean (SD), median (min–max) for the numeric and non-normal variables, and frequency (percentages) for categorical variables. A paired *t* test and Wilcoxon signed-rank test was used to compare continuous variables within groups. McNemar was used to compare categorical variables within groups. Comparison between groups for continuous variables was made by repeated-measure analysis of variance using a mixed model after transformation of the data to enhance normality, with treatment, time (categorical), and their interaction as fixed effects and with random patient effects. In addition, data at each time point were compared with independent samples *t* tests, Mann–Whitney *U* test, and Chi-square test depending on the variables. All *p* values were two-tailed and statistical significance was taken as a *p* value of less than 0.05. Multiple imputation for incomplete records was not needed, because less than 5% of the data was missing. An interim analysis was not performed for this study.

## Results

**Fig. 1:**
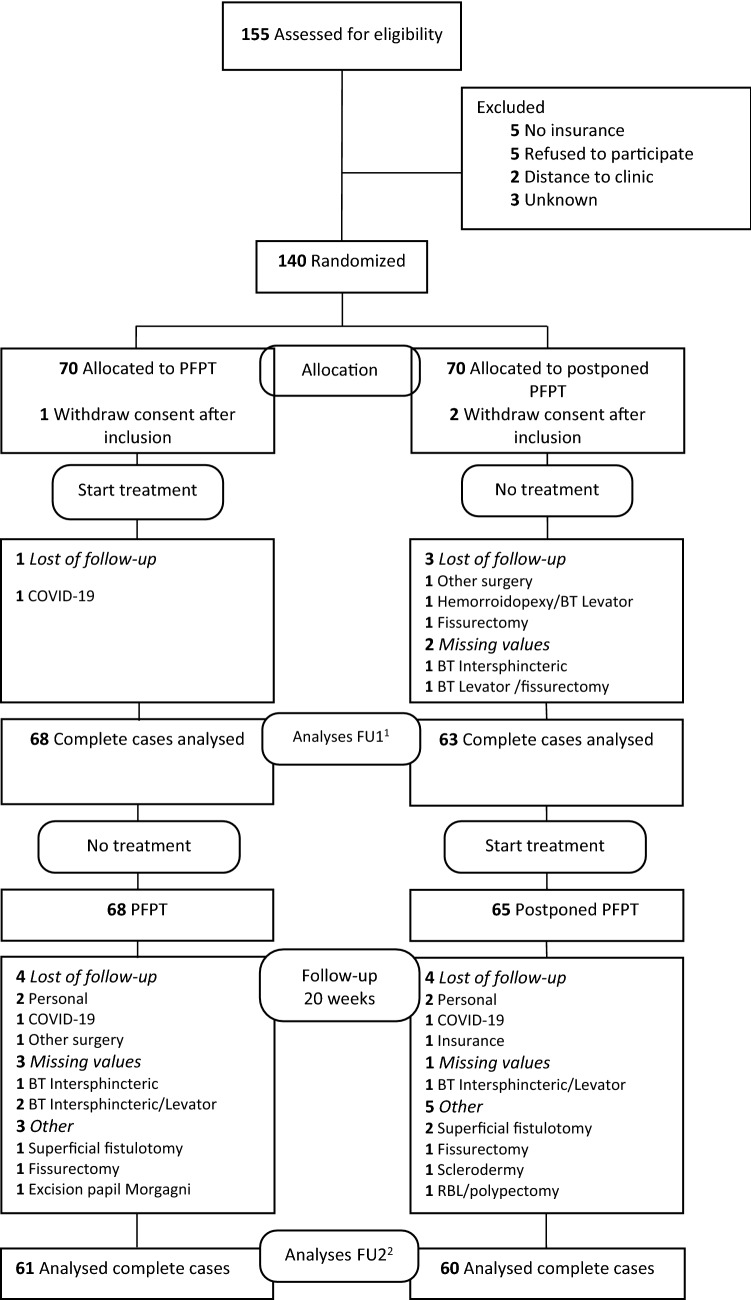
CONSORT diagram. 1 Timepoint 8 weeks after inclusion; 2 Timepoint 20 weeks after inclusion. *PFPT* Pelvic Floor Physical Therapy, *BT* Botulinum Toxin, *RBL* Rubber Band Ligation, *FU* Follow-up

Between 10 December 2018 and 13 July 2021, 155 patients with CAF were found eligible. 140 patients, 68 men (48.6%) and 72 women (51.4%) with a mean age of 44.5 ± 11.1 (range 19–79) years were randomized to PFPT (*n* = 70) or a control group (postponed PFPT) (*n* = 70). Baseline characteristics were similar between the 2 groups (Table 1). After randomization, one patient in the PFPT group and 2 patients in the control group withdrew after inclusion. During the study, 4 patients were lost of follow-up at 8 weeks, one patient in the PFPT group and 3 in the control group. At 20 weeks after inclusion, 4 patients were lost of follow-up in the PFPT- group and 4 in the control group (Fig. 1: CONSORT diagram).

**Table 1:** Baseline characteristics according to treatment group

Variable	PFPT group (*n* = 70)	Control (postponed PFPT) (*n* = 70)
Age, years mean ± SD,(range)	44.2 ± 10.7,(23–66)	44.7 ± 11.6,(19–79)
Sex, women/men, *n* (%)	37(52.9)/33(47.1)	35(50.0)/35(50.0)
Partus, yes/no (%) Vaginal/C-section (%)	31.4/21.4 28.6/2.9	30/20 25.7/4.3
Duration of complaints (%) 0–2 months 2–6 months 6–12 months 12–36 months > 3 years	12.9 18.6 12.9 24.3 31.4	11.4 27.1 15.7 20.0 25.7
Smoking, yes/no (%)	7.1/92.9	11.4/88.6
Gastric bypass, yes/no (%)	2.9/97.1	4.3/95.7
Previous treatment; Botulinum toxin, yes/no (%) Lateral internal sphincterotomy, yes/no (%) Alternate, yes/no (%)	10/90 1.4/98.6 37.1/62.9	5.7/94.3 0.0/100 32.9/67.1
Obstipation, yes/no (%)	12.9/87.1	17.1/82.9
Use of laxatives/fiber, yes/no (%)	44.3/55.7	47.1/52.9
Sexual complaints, yes/no (%)	27.1/72.9	24.3/75.7
Psychological consultant, yes/no (%)	37.1/62.9	27.1/72.9
Urological complaints, yes/no (%)	25.7/74.3	28.6/71.4
Location of fissure (%)		
Anterior	12.9	15.7
Posterior	78.6	77.1
Other	8.6	7.1
Anal sphincter pressure (%)		
Decreased	1.4	1.4
Normal	12.9	10,0
Increased	85.7	88,6
Pelvic floor resting tone (%)		
Decreased	2.9	4.3
Normal	10.0	15.7
Increased	87.1	80.0
Squeeze pressure (%)		
Decreased	34.3	31.4
Normal	48.6	50.0
Increased	17.1	18.6
Traction puborectalis painful, yes/no (%)	70/30	80/20
Dyssynergia digital rectal examination, yes/no (%)	67.1/32.9	78.6/21.4
Proctoscopy, yes/no (%)	45.7/54.3	42.9/57.1
Ointment (%)		
Diltiazem	94.3	88.6
Isosorbine dinitrate (ISDN)	4.3	10.0
Other	1.4	1.4

There were no reported negative side-effects or serious adverse events in both groups.

### Primary outcome

**Fig. 2:**
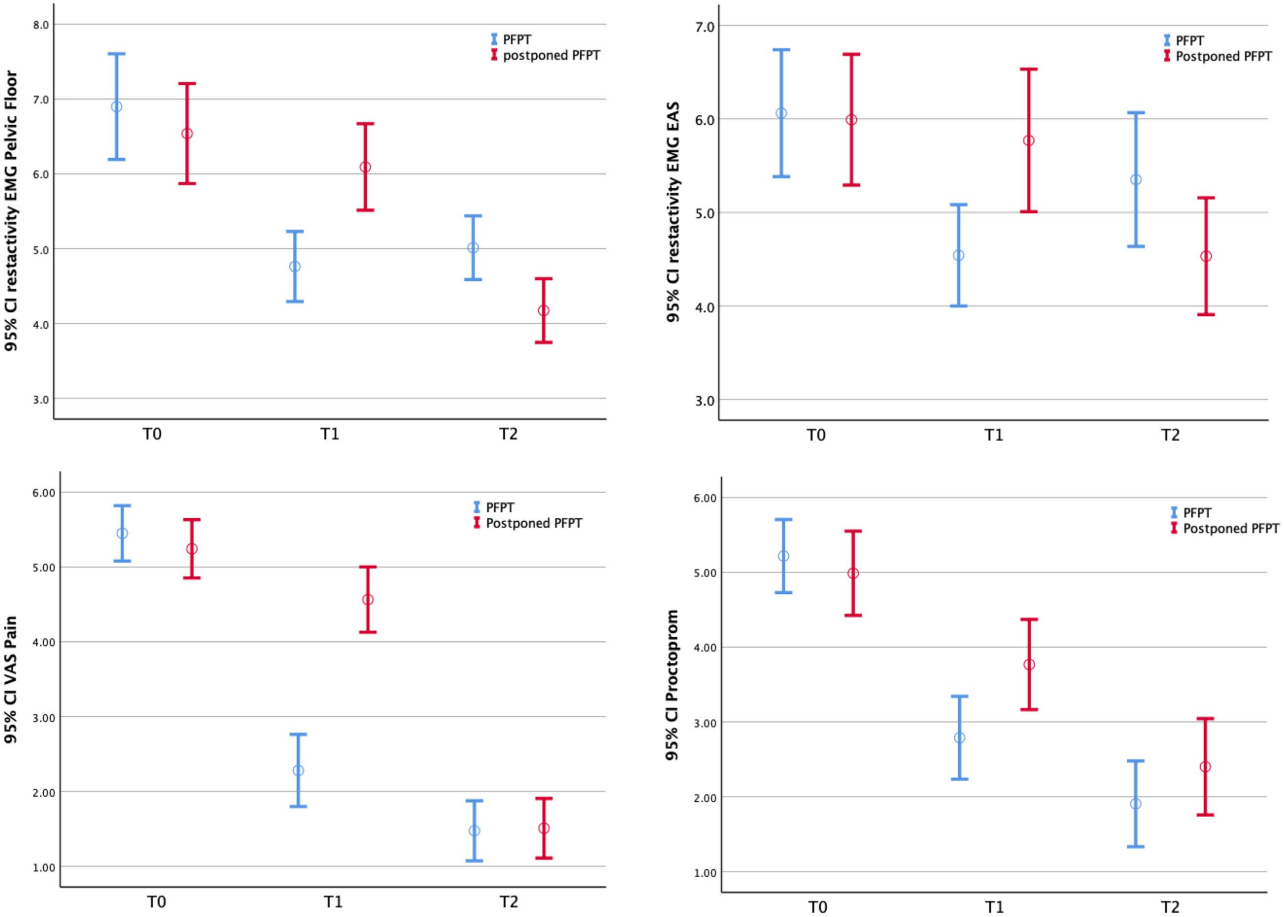
Repeated measurement analyses. *VAS* Visual Analogue Scale, *EMG* Electromyography, *PFPT* Pelvic Floor Physical Therapy, *CI* Confidence Interval

Regarding the analysis of repeated measures, the PFPT group was found to be more effective for reducing pelvic floor muscle tone measured with EMG compared to the control group (*p* < 0.001) (Fig. 2; Table 2). The mean estimated difference between groups post-treatment at first follow-up, at 8 weeks from baseline was − 1.88 µV; 95% CI − 2.49 to -− 1.27 (*p* < 0.001). At 20 weeks, when both groups had received PFPT, the mean difference between the PFPT group and control group showed no significance (− 0.05 µV; 95% CI − 0.82 to 0.71; *p* = 0.889) (Table 2).

**Table 2:** Baseline characteristics according to treatment group

	PFPT group					Control group					Between groups 8 weeks	Between groups 20 weeks	Group vs Time
	Baseline	8 weeks	*p* value	20 weeks	*p* value	Baseline	8 weeks	*p* value	20 weeks	*p* value	*p* value	*p* value	*p* value
EMG PF resting tone (μV), mean (SD)	6.9(2.9)	4.8(1.9)	< 0.001^*^	5.0(1.7)	< 0.001^*^	6.5(2.8)	6.1(2.3)	0.192^*^	4.2(1.7)	< 0.001^*^	< 0.001^§^	0.889^§^	< 0.001^@^
EMG EAS resting tone (μV), mean (SD)	6.1(2.8)	4.5(2.1)	< 0.001^*^	5.4(2.7)	< 0.05^*^	6.0(2.8)	5.8(2.7)	0.173^*^	4.5(2.3)	< 0.001^*^	< 0.05^§^	0.331^§^	< 0.001^@^
Fissure healed yes (%)	0.0	55.7	< 0.001^ψ^	55.7	< 0.001^ψ^	0.0	21.4	< 0.001^ψ^	60.0	< 0.001^ψ^	< 0.001^χ^	0.333^χ^	NA
VAS pain score, mean (SD)	5.5(1.6)	2.3(1.9)	< 0.001^*^	1.5(1.6)	< 0.001^¢^	5.2(1.6)	4.6(1.8)	< 0.001^*^	1.5(1.6)	< 0.001^¢^	< 0.001^§^	0.425^α^	< 0.001^@^
Increased tone PF (%)	87.1	28.6	< 0.001^ψ^	22.9	< 0.001^ψ^	81.4	77.1	0.980^ψ^	20.0	< 0.001^ψ^	< 0.05^χ^	0.750^χ^	NA
Proctoprom mean (SD)	5.2(2.0)	2.8(2.1)	< 0.001^¢^	1.9(1.9)	< 0.001^¢^	5.0(2.2)	3.8(2.2)	< 0.05^¢^	2.4(2.1)	< 0.001^¢^	< 0.001^α^	0.118^α^	< 0.001^@^
Dyssynergia DRE yes (%)	67.1	25.7	< 0.001^ψ^	24.3	< 0.001^ψ^	78.6	64.3	0.092^ψ^	22.9	< 0.001^ψ^	< 0.001^χ^	0.964^χ^	NA
Dyssynergia BET yes (%)	38.6	NA	NA	5.7	< 0.001^ψ^	45.7	NA	NA	4.3	< 0.001^ψ^	NA	0.566^χ^	NA

Proctoprom sample sizes are 64 and 61 PFPT and control respectively at baseline, 58 and 54 respectively at 8 weeks follow-up and 44 and 45 respectively at 20 weeks follow up Dyssynergia BET sample sizes are 34 and 35 for PFPT and control respectively. At 20 weeks sample sizes are 18 PFPT vs 20 control. PFPT=Pelvic Floor Physical Therapy; EMG=Electromyography; EAS=External Anal Sphincter; VAS=Visual Analog Scale; NA= not applicable; PF=Pelvic Floor; DRE= Digital Rectal Examination; BET=Balloon Expulsion Test; PF=Pelvic Floor

^*^Paired t-test, comparison of scores between baseline and 8 weeks and 20 weeks

^§^Unpaired t-test comparison of change scores from baseline to week 8 and 20 weeks

^@^Repeated measurement analyses

^¢^Wilcoxon signed rank test

^α^Mann-Whitney* U* test

^ψ^McNemar

^χ^Chi-square test

^¥^The sample sizes shown are slightly smaller for some secondary outcomes due to missing values.

The mean tone of the pelvic floor at rest measured with EMG, decreased significantly from pre- to post-treatment in the PFPT- group (*p* < 0.001) and remained significant from baseline to 20-week follow-up (*p* < 0.001) (Table 2). In the control group, the mean resting tone of the pelvic floor did not decrease significantly at first follow-up (*p* = 0.192). At 20-week follow-up, the control group showed a significant decrease in mean resting tone of the pelvic floor after treatment (*p* < 0.001) (Table 2).

Regarding the analysis of repeated measures, treatment in the PFPT group was found to be more effective for reducing EAS-tone (measured with EMG), compared to the control group (*p* < 0.001) (Fig. 2; Table 2). The mean estimated difference between groups after treatment was − 1.44 µV; 95% CI − 2.77 to − 0.12 (*p* < 0.05). At 20 weeks, no significant difference was found between groups (0.61 µV; 95% CI − 0.62 to 1.84; *p* = 0.331) (Table 2).

The mean resting tone of the EAS in the PFPT- group, decreased significantly from pre- to post-treatment (*p* < 0.001) and remained significant at 20-week follow-up (*p* < 0.05). No significant decrease was found in the mean resting tone of the EAS at first follow-up in the control group (*p* = 0.173). After intervention at 20-week follow-up, the mean resting tone of the EAS decreased significantly in the control group (*p* < 0.001) (Table 2).

### Secondary outcomes

#### Clinical healing of the fissure

In the PFPT group, the fissure was healed in 55.7% of the patients vs 21.4% in the control group at 8 week follow-up (*p* < 0.001). At 20-week follow-up, healing of the fissure did not further improve in the PFPT group, but was healed in 60% in the control group after treatment (*p* < 0.001). No significant differences were found in fissure healing between groups at 20-week follow-up (*p* = 0.333) (Table 2).

#### Pain

Regarding the analysis of repeated measures, it was found that the VAS pain score was more effectively reduced in the PFPT group compared to the control group (*p* < 0.001) (Fig. 2, Table 2). The mean estimated difference between groups at 8 weeks from baseline was − 2.47; 95% CI − 3.05 to − 1.89 (*p* < 0.001). At 20 weeks, no significant mean difference in VAS pain scores was found between groups (− 0.17; 95% CI − 0.89 to 0.54; *p* = 0.425) (Table 2).

VAS pain was significantly reduced in both the PFPT and the control group at 8 weeks from baseline (*p* < 0.001). At 20-week follow-up, the VAS pain score in the PFPT-group and control group further decreased and remained significant compared to baseline (*p* < 0.001) (Table 2).

#### Pelvic floor function

Dyssynergia measured with digital rectal examination was found in 67.1% in the PFPT group vs 78.6% in control group before treatment. After intervention at 8 weeks from baseline, dyssynergia was found in 25.7% in the PFPT group vs in 64.3% in control group (*p* < 0.001). At 20-week follow-up, when both groups received treatment, the difference in dyssynergia was no longer significant between groups (*p* = 0.964) (Table 2).

At baseline, dyssynergia measured with the balloon expulsion test was found in 38.6% in PFPT group vs 45.7% in control group. After 20 weeks, no significance was found in dyssynergia measured with the balloon expulsion test in the PFPT group vs the control group (*p* = 0.566) (Table 2).

Increased pelvic floor muscle tone measured with digital rectal examination was found in 87.1% of the patients in the PFPT group vs 81.4% in control group before treatment. After intervention at 8 weeks from baseline, increased pelvic floor muscle tone was found in 28.6% in the PFPT group vs 77.1% in the control group (*p* < 0.05). At 20-week follow-up, no significance was found in increased pelvic floor muscle tone between the two groups after treatment (*p* = 0.750) (Table 2).

#### Patient-related outcome measurement

According to repeated measurement analysis, complaints were more effectively reduced in the PFPT group compared to the control group at 8 weeks from baseline (*p* < 0.001) (Fig. 2; Table 2). The mean estimated difference between groups at 8 weeks from baseline was − 1.56; 95% CI − 2.24 to − 0.88 (*p* < 0.001). At 20 weeks, no significant difference in Proctoprom scores was found between groups (− 0.66; 95% CI − 1.59 to 0.28; *p* = 0.118) (Table 2).

The Proctoprom scores in the PFPT-group decreased significantly from pre- to post-treatment at 8 weeks from baseline (*p* < 0.001). In the control group, the Proctoprom scores also decreased (*p* < 0.05). Improvement of Proctoprom scores were maintained in both groups at 20-week follow-up (*p* < 0.001) (Table 2).

## Discussion

The present study is the first randomized clinical trial of EMG-biofeedback-assisted PFPT for CAF. The results of our study show a significant decrease in mean resting tone of the pelvic floor measured with digital rectal examination and EMG, improvement of healing of the fissure, pelvic floor function, pain, and complaint reduction. These results confirm our hypothesis that PPFT is effective in patients with CAF.

Pelvic floor muscle tone measured with EMG-biofeedback decreased from pre-to post-treatment and between groups and has been proven an effective and efficient treatment modality. Biofeedback is a neuromuscular training approach in which patients learn how to appropriately contract or relax muscles, aided by visual or auditory feedback of muscle activity. It is the mainstay in the treatment of anorectal dysfunctions and is commonly utilized in PFPT (35]. The efficacy of PFPT including biofeedback on pelvic floor dysfunction has already been proven in randomized control trials [19, 36, 37], although the success depends on motivation of the patient and skills of the therapist [22].

Muscle tone measured with EMG, also improved in the EAS from pre- to post-treatment and compared to controls. These results confirm the role of the EAS in patients with CAF, which correlates with findings of Grimaud [38]. In this study, including patients with chronic idiopathic anal pain, biofeedback was used for relaxation of the EAS. A significant decrease in resting pressure was observed in the anal canal measured with manometry, which was accompanied by a relief in anal pain, suggesting that the pain due to abnormal chronic contraction of the EAS.

Pelvic floor muscle tone, based on digital rectal examination significantly decreased from pre- to post-treatment and between groups. A comprehensive careful digital rectal examination is an important topic to obtain information on anorectal anatomy and function [22, 26]. Besides that, the use of quantified digital palpation to measure muscle tone and dyssynergia is recommended in clinical guidelines [4, 25]. Although no normative values on pelvic floor muscle tone exits, it appears that patients with CAF have higher levels of tonic activation of the pelvic floor. Furthermore, tenderness to palpation often accompanied with increased pelvic floor muscle tone is a feature of levator ani syndrome [4, 39] and was found in 75% of our patients. Increased tone or spasm of the levator ani, probably leading tot ischemia could be a contributing factor in the pain patients experience [40]. Tenderness to palpation is a predicting factor of response to biofeedback treatment [41].

Fourteen percent of the fissures were anterior, mainly in women (70%), 35% of whom had had a vaginal delivery. Anterior fissures are associated with low anal sphincter pressure in the presence of anal sphincter defects [42], but a subgroup analysis showed high anal sphincter pressure in 90% of these women. In contrast, high anal sphincter pressure was found in 87% of posterior fissures. This outcome is quite interesting, although it should be mentioned that we investigated anal sphincter pressure with digital rectal examination and not with manometry. The presence of pain and an alteration of anal sensibility [43] could blur correct anal sphincter pressure and result in a higher pressure. Several studies about comparison between digital rectal examinations show an overall good agreement in pressures with manometry, but the results are not consistent [43–47]. These results should be interpreted with care.

Dyssynergia of the pelvic floor was found in a large percentage (72,9%) of our patients at baseline. Subgroup analyses showed less dyssynergia (56%) in patients with low/normal pressures compared to patients with high anal sphincter pressures (76%). This is comparable to the study of Jain et al.[48], in which 426 patients with fecal evacuation disorders were investigated with anorectal manometry. Dyssynergia was more common in patients with CAF. Whether CAF is secondary to dyssynergic defecation or responsible for an abnormal defecation pattern is still under debate.

Treatment with biofeedback for dyssynergia is highly recommended in clinical guidelines [4, 23] and was also successful in our study, considering the improvement in dyssynergic pattern of the pelvic floor after treatment, although 22% of the patients did not improve.

Dyssynergia is affected by alterations of the chest, abdominal wall, and vertebral column and pelvic floor that may be functional, anatomical or behavioral and  which may influence the outcome of PFPT [20, 49]. It is important to perform a comprehensive evaluation of these alterations with a multidimensional approach to define which patients will benefit most from PFPT [50].

The Proctoprom was used to detect changes over time, the patient’s state of health measures, and the effect of treatment [31]. This study showed a significant effect of disease burden and treatment from the patient point of view.

Although the PFPT group improved in all the outcome measures, patients in the control group also improved significantly regarding pain and Proctoprom scores, at first follow-up. The first step in treatment is re-education and understanding defecation disorders [51]. Probably, the information all patients receive about their complaint, instruction about toilet behavior, and lifestyle advice contribute to this improvement.

An evident decrease of pelvic floor muscle tone, improvement of fissure healing, and pelvic floor function at 20-week follow-up indicated that patients from the postponed PFPT group also benefited from PFPT. Although patients from the early PFPT group improved quickly, it is still worthwhile initiating PFPT at any time during treatment.

The main strengths of this study are the prospective randomized control trial design, sufficiently powered intent-to treat analyses and the design of the study in which all patients received PFPT. In addition, the use of a PFPT- protocol performed by large group of collaborating pelvic floor physical therapist in the Netherlands makes this treatment suitable in all clinical settings. All pelvic floor physical therapists involved in the study were highly trained and had access to equipment for EMG-biofeedback. The use of a validated EMG electrode [27] to measure pelvic floor muscle tone, the use of a standardized measurement protocol by the same investigator in the same environment diminished information bias [52].

The willingness to participate and adherence of the patients to the trial procedures and the intervention was high, evidenced by the low rate of loss of follow-up. The use of this clinical trial set up with a postponed PFPT- group may have also positively influenced the adherence rate. Patients knew they would start with PFPT, albeit 8 weeks later.

Our population was real world; we enrolled patients of all ages and both sexes with duration of complaints varying from 2 months to more than 3 years and living in different parts of the Netherlands Thus, the results may be generalizable to the CAF population at large.

There were several limitations in our study. The first concerns the risk of detection bias; we were unable to mask group allocation from patients, collaborating pelvic floor physical therapist and principal investigator, because of the trial design and the nature of the intervention. Second, the pelvic floor physical therapist was also the principal investigator and consequently investigator’s bias could not be ruled out.

The balloon expulsion test, to identify patients with pelvic floor dyssynergia was only performed in 69 patients at inclusion with a high rate of loss to follow-up at 20 weeks. The main reason was a logistic one. It was not always possible to combine an appointment in the clinic with the nurse and principal investigator, especially during the COVID-19 pandemic. In addition, in a large percentage the balloon expulsion test failed. This could be a result of fear of patients with CAF in expelling a balloon.

COVID-19 did have some influence on our study. During the first pandemic in 2020, we were not able to include patients in the study for 4 months and a small number of patients were lost to follow-up, because they were diagnosed with COVID-19 at the follow-up appointment.

Clinical guidelines of leading societies do not recommend PFPT as a treatment option for CAF. Our findings provide strong evidence that PFPT is effective in the treatment of CAF and pelvic floor dysfunction. PFPT has no side-effects, low potential for complications, and low costs.

## Conclusions

Our findings confirm that PFPT is effective in patients with CAF and concomitant pelvic floor dysfunction in improving pelvic floor muscle tone and function, healing of the fissure, reducing pain, and complaint reduction. This study provides evidence that PFPT can be used as adjuvant treatment in CAF and pelvic floor dysfunction besides regular conservative treatment.

## References

[1] Griffin N, Acheson AG, Tung P, Sheard C, Glazebrook C, Scholefield JH (2004). Quality of life in patients with chronic anal fissure. Colorectal Dis.

[2] Arısoy Ö, Şengül N, Çakir A (2016). Stress and psychopathology and its impact on quality of life in chronic anal fissure (CAF) patients. Int J Colorectal Dis.

[3] Nelson RL, Thomas K, Morgan J, Jones A (2012). Non surgical therapy for anal fissure. Cochrane Database Syst Rev.

[4] Wald A, Bharucha AE, Limketkai B, Malcolm A, Remes-Troche JM, Whitehead WE (2021). ACG clinical guidelines: management of benign anorectal disorders. Am J Gastroenterol.

[5] Schouten WR, Briel JW, Fau-Auwerda JJ, Auwerda JJ (1994). Relationship between anal pressure and anodermal blood flow. The vascular pathogenesis of anal fissures. Dis colon Rectum.

[6] van Meegdenburg MM, Trzpis M, Heineman E, Broens PM (2016). Increased anal basal pressure in chronic anal fissures may be caused by overreaction of the anal-external sphincter continence reflex. Med Hypotheses.

[7] Farouk R, Duthie GS, MacGregor AB, Bartolo DC (1994). Sustained internal sphincter hypertonia in patients with chronic anal fissure. Dis Colon Rectum.

[8] Lund JN, Scholefield JH (1996). Aetiology and treatment of anal fissure. Br J Surg.

[9] Palit S, Lunniss PJ, Scott SM (2012). The physiology of human defecation. Dig Dis Sci.

[10] Rao SS, Welcher KD, Leistikow JS (1998). Obstructive defecation: a failure of rectoanal coordination. Am J Gastroenterol.

[11] Ooijevaar RE, Felt-Bersma RJF, Han-Geurts IJ, van Reijn D, Vollebregt PF, Molenaar CBH (2019). Botox treatment in patients with chronic functional anorectal pain: experiences of a tertiary referral proctology clinic. Tech Coloproctol.

[12] Andrews CN, Storr M (2011). The pathophysiology of chronic constipation. Can J Gastroenterol.

[13] Steele SR, Madoff RD (2006). Systematic review: the treatment of anal fissure. Aliment Pharmacol Ther.

[14] Boland PA, Kelly ME, Donlon NE, Bolger JC, Larkin JO, Mehigan BJ (2020). Management options for chronic anal fissure: a systematic review of randomised controlled trials. Int J Colorectal Dis.

[15] Sahebally SM, Meshkat B, Walsh SR, Beddy D (2018). Botulinum toxin injection vs topical nitrates for chronic anal fissure: an updated systematic review and meta-analysis of randomized controlled trials. Colorectal Dis.

[16] Nelson RL, Manuel D, Gumienny C, Spencer B, Patel K, Schmitt K (2017). A systematic review and meta-analysis of the treatment of anal fissure. Tech Coloproctol.

[17] Garg P, Garg M, Menon GR (2013). Long-term continence disturbance after lateral internal sphincterotomy for chronic anal fissure: a systematic review and meta-analysis. Colorectal Dis.

[18] Arroyo A, Perez F, Serrano P, Candela F, Lacueva J, Calpena R (2005). Surgical versus chemical (botulinum toxin) sphincterotomy for chronic anal fissure: long-term results of a prospective randomized clinical and manometric study. Am J Surg.

[19] van Reijn-Baggen DA, Han-Geurts IJM, Voorham-van der Zalm PJ, Pelger RCM, Hagenaars-van Miert C, Laan ETM. Pelvic Floor Physical Therapy for Pelvic Floor Hypertonicity: A Systematic Review of Treatment Efficacy. LID S2050–0521(21)00012–3 10.1016/j.sxmr.2021.03.002. 2021(2050–0521 (Electronic)). 10.1016/j.sxmr.2021.03.002 34127429

[20] Bocchini R, Chiarioni G, Corazziari E, Pucciani F, Torresan F, Alduini P (2019). Pelvic floor rehabilitation for defecation disorders. Tech Coloproctol.

[21] Chiarioni G, Heymen S, Fau-Whitehead W-E, Whitehead WE (2006). Biofeedback therapy for dyssynergic defecation. World J Gastroenterol.

[22] Patcharatrakul T, Rao SSC (2018). Update on the pathophysiology and management of anorectal disorders. Gut Liver.

[23] Rao SS, Benninga M, Fau-Bharucha AE, Bharucha AF, Chiarioni G, Chiarioni G, Fau-Di LC, Di Lorenzo C, Fau-Whitehead WE, Whitehead WE (2015). ANMS-ESNM position paper and consensus guidelines on biofeedback therapy for anorectal disorders. Neurogastroenterol Motil.

[24] Lee HJ, Boo SJ, Jung KW, Han S, Seo SY, Koo HS (2015). Long-term efficacy of biofeedback therapy in patients with dyssynergic defecation: results of a median 44 months follow-up. Neurogastroenterol Motil.

[25] Frawley H, Shelly B, Morin M, Bernard S, Bo K, Digesu GA (2021). An International Continence Society (ICS) report on the terminology for pelvic floor muscle assessment. Neurourol Urodyn.

[26] Rao SSC (2018). Rectal Exam: Yes, it can and should be done in a busy practice!. Am J Gastroenterol.

[27] Voorham-van der Zalm PJ, Voorham Jc Fau - van den Bos TWL, van den Bos Tw Fau - Ouwerkerk TJ, Ouwerkerk Tj Fau - Putter H, Putter H Fau - Wasser MNJM, Wasser Mn Fau - Webb A, et al. Reliability and differentiation of pelvic floor muscle electromyography measurements in healthy volunteers using a new device: the Multiple Array Probe Leiden (MAPLe). Neurourol Urodyn. 2013 32:341–8. 10.1002/nau.22311 22972554

[28] Tantiphlachiva K, Rao P, Fau-Attaluri A, Attaluri A, Fau-Rao SSC, Rao SS (2010). Digital rectal examination is a useful tool for identifying patients with dyssynergia. Clin Gastroenterol Hepatol.

[29] Chiarioni G, Kim SM, Vantini I, Whitehead WE (2014). Validation of the balloon evacuation test: reproducibility and agreement with findings from anorectal manometry and electromyography. Clin Gastroenterol Hepatol.

[30] Dworkin RH, Turk DC, Wyrwich KW, Beaton D, Cleeland CS, Farrar JT (2008). Interpreting the clinical importance of treatment outcomes in chronic pain clinical trials: IMMPACT recommendations. J Pain.

[31] Vander MGJ, Molenaar C, Buyl R, Westert G, van der Wees PJ. How is your proctology patient really doing? Outcome measurement in proctology: development, design and validation study of the Proctoprom. Tech Coloproctol. 2020:291–300. 10.1007/s10151-020-02156-2 32112248

[32] Reijn-Baggen DAv, H.W.Elzevier, R.C.M.Pelger, I.J.M.Han-Geurtsa. Pelvic floor physical therapy in the treatment of chronic anal fissure (PAF-study): Study protocol for a randomized controlled trial. Contemporary Trials Communications. 2021;24. 10.1016/j.conctc.2021.100874 PMC8606324 34841124

[33] Dodi G, Bogoni F, Bogoni F, Infantino A, Pianon P, Mortellaro LM, Lise M (1986). Hot or cold in anal pain? A study of the changes in internal anal sphincter pressure profiles. Dis Colon Rectum.

[34] Castor. [Available from: https://www.castoredc.com.

[35] Bharucha AE, Lacy BE (2020). Mechanisms, evaluation, and management of chronic constipation. Gastroenterology.

[36] Chiarioni G, Nardo A, Vantini I, Romito A, Whitehead WE, Whitehead WE (2005). Biofeedback is superior to electrogalvanic stimulation and massage for treatment of levator ani syndrome. Dis Colon Rectum.

[37] Heymen S, Scarlett Y, Jones K, Jones K, Ringel Y, Drossman D, Whitehead WE, Whitehead WE (2007). Randomized, controlled trial shows biofeedback to be superior to alternative treatments for patients with pelvic floor dyssynergia-type constipation. Dis Colon Rectum.

[38] Grimaud JC, Bouvier M, Naudy B, Guien C, Salducci J (1991). Manometric and radiologic investigations and biofeedback treatment of chronic idiopathic anal pain. Dis Colon Rectum.

[39] Bharucha AE, Lee TH (2016). Anorectal and Pelvic Pain. Mayo Clin Proc.

[40] Everaert K, Devulder J, De Muynck M, Stockman S, Depaepe H, De Looze D (2001). The pain cycle: implications for the diagnosis and treatment of pelvic pain syndromes. Int Urogynecol J Pelvic Floor Dysfunct.

[41] Chiarioni G, Nardo A, Vantini I, Romito A, Whitehead WE, Whitehead WE (2010). Biofeedback is superior to electrogalvanic stimulation and massage for treatment of levator ani syndrome. Gastroenterology.

[42] Jenkins JT, Urie A, Molloy RG (2008). Anterior anal fissures are associated with occult sphincter injury and abnormal sphincter function. Colorectal Dis.

[43] Beatrice D, Gaetano DV, Dario C, Girolamo G. Reliability of digital rectal examination as compared to anal manometry in chronic anal fissure. 2021(0219–3108 (Electronic)):1021–2. 10.1016/j.asjsur.2021.04.044 34052084

[44] Dobben AC, Terra MP, Deutekom M, Gerhards MF, Bijnen AB, Felt-Bersma RJ (2007). Anal inspection and digital rectal examination compared to anorectal physiology tests and endoanal ultrasonography in evaluating fecal incontinence. Int J Colorectal Dis.

[45] Soh JS, Lee HJ, Jung KW, Yoon IJ, Koo HS, Seo SY (2015). The diagnostic value of a digital rectal examination compared with high-resolution anorectal manometry in patients with chronic constipation and fecal incontinence. Am J Gastroenterol.

[46] Felt-Bersma RJ, Klinkenberg-Knol EC, Meuwissen SG. Anorectal function investigations in incontinent and continent patients. Differences and discriminatory value. Dis Colon Rectum. 1990;33(6):479–85 **(discussion 85–6)**. 10.1007/BF02052142 2351000

[47] Eckardt VF, Kanzler G. How reliable is digital examination for the evaluation of anal sphincter tone? (0179–1958 (Print)). 10.1007/BF00299335 8409694

[48] Jain M, Baijal R, Srinivas M, Venkataraman J (2019). Fecal evacuation disorders in anal fissure, hemorrhoids, and solitary rectal ulcer syndrome. Indian J Gastroenterol.

[49] Brusciano L, Gualtieri G, Gambardella C, Terracciano G, Tolone S, Del Genio G (2020). Pelvic floor dyssynergia: the new iceberg syndrome. Tech Coloproctol.

[50] Brusciano LA-O, Gambardella CA-O, Del Genio GA-O, Tolone SA-O, Lucido FA-O, Terracciano GA-O, et al. Outlet obstructed constipation and fecal incontinece: is rehabilitation treatment the way? Myth or reality. Arq Gastroenterol 2020;5757(1678–4219 (Electronic)):198–202. 10.1590/S0004-2803.202000000-38 32401951

[51] Beaty JS, Shashidharan M (2016). Anal fissure. Clin Colon Rectal Surg.

[52] McLean L, Brooks K (2017). What does electromyography tell us about dyspareunia?. Sexual Med Rev.

